# Time in Therapeutic Range in Patients with Venous Thromboembolism on Vitamin K Antagonist in a Moroccan Thrombosis Unit : An Observational Study

**DOI:** 10.1055/a-2890-0814

**Published:** 2026-06-19

**Authors:** Zoubida Tazi Mezalek, Wafaa Mounfaloti, Fatima Zahra Iraqi, Wafaa Ammouri, Hicham Harmouche, Mouna Maamar, Rachid Razine

**Affiliations:** 1 Department of Internal Medecine/Clinical Hematology 479557 Ibn Sina University Hospital CenterRabat, Rabat-Sale-Zemmour-ZaerMorocco; 2 97980 Mohammed V University of Rabat Faculty of Medicine and Pharmacy of RabatRabat, Rabat-Sale-Zemmour-ZaerMorocco; 3 Laboratory of Biostatistics, Epidemiology and Clinical Research, Faculty of Medicine and PharmacyRabat, Rabat-Sale-Zemmour-ZaerMorocco

**Keywords:** venous thromboembolism, vitamin K antagonist, time in therapeutic range

## Abstract

**Background:**

Patients with venous thromboembolism (VTE) frequently require vitamin K antagonists (VKAs) to treat and prevent recurrent events.

**Aims:**

We performed a study to assess the quality of anticoagulation in patients with VTE treated with VKAs using time in therapeutic range (TTR), to identify associated factors with predictors of poor control and outcomes in VTE patients.

**Methods:**

We performed an observational, retrospective study that included VTE patients treated with VKA. The primary outcome was the TTR calculated according to the Rosendaal algorithm.

**Results:**

A total of 171 VKA-treated patients were evaluated (53.2% men). The average age of our patients at diagnosis was 49.8 + 16.6 years [17–92]. VTE's most common location was the lower limbs, with 91.8% cases, proximal in 83.6%. Patients were followed for a mean period of 178 days. The mean TTR was 41.2% (standard deviation: 32.9%), and 74.3% of the patients had a mean TTR < 65%. The rates of major bleeds and thromboembolic events were 0.5 and 2.2%, respectively. After multivariate statistical analysis, age older than 65 and surgery provoked VTE were associated with better TTR.

**Conclusions:**

These results are informative and may help identify patients who require closer monitoring or innovative strategies to optimize oral anticoagulant therapy outcomes.

## Introduction

Venous thromboembolism (VTE) is a major public health problem, and early diagnosis and effective treatment are critical for preventing further complications and adverse prognosis. Vitamin K antagonists (VKAs) are still the most commonly prescribed oral anticoagulants in resource-limited countries. Alternative direct oral anticoagulants (DOACs) are not routinely available in public sector health care facilities owing to their high cost.

The safety and effectiveness of VKAs depend greatly on the proportion of time patients spend within the international normalized ratio (INR) therapeutic range. The absence of standard dosages of VKA makes it imperative to perform serial INR tests and to monitor the quality of anticoagulation. Time in therapeutic range (TTR) is a widely accepted metric used to evaluate the quality of VKA therapy. 1 2 While TTR ≥ 65% is considered good, patients with lower TTR are at higher risk of poor outcomes, 3 4 5 and current antithrombotic guidelines recommend interventions for improving low TTR. 6 Many studies have primarily explored the quality of anticoagulation in patients with atrial fibrillation (AF), whereas in patients with VTE, it has scarcely been investigated and variables altering the TTR in patients with VTE have yet to be clearly determined.

INR control is often poor in low-income countries. There are no reports in the literature regarding TTR values in Moroccan patients, and this is the first study that evaluates TTR in this setting. Therefore, we conducted this present study in one of the most frequently visited university hospitals in the capital city of Rabat. We analyzed factors related to the quality of anticoagulation in patients with VTE undergoing oral anticoagulation treatment with VKAs.

## Methods

### Study Design and Participants

We conducted a retrospective, single-center cohort study and collected all data by reviewing medical records and accessing the patient history of anticoagulation treatment for VTE. The Human Research Ethics Committee at the University Faculty of Medicine and Pharmacy in Rabat approved the study.

Participants needed to fulfill the following criteria: age ≥ 18 years, confirmed diagnosis of VTE (deep vein thrombosis [DVT] or pulmonary embolism [PE]), and prescription of anticoagulation therapy with VKA for at least 6 months. We also included patients with at least 3 INR values. The set of variables and clinical parameters included sex, age, duration of treatment, primary diagnosis (DVT, PE, or both), and comorbidities (hypertension, diabetes, hyperlipidemia, previous or current cancer, renal impairment, any vascular disease, thrombophilia or inflammatory disease, and smoking habit). Patients were clustered into subgroups according to their age, sex, and etiology of VTE.

We calculated the percentage of TTR with computer software per the Rosendaal method. 7 In the present study, we established the cutoff for high-quality anticoagulation at TTR ≥ 65%, in accordance with the recommendations. 6

### Statistics

All analyses were conducted using SPSS software (v. 13.0). Categorical variables are expressed as number and percentage. Quantitative variables were expressed as mean ± standard deviation (SD) or median (interquartile range). We used descriptive statistics to examine all variables and clinical parameters. To evaluate factors potentially associated with poor anticoagulation control (TTR < 65%), we conducted a univariate comparative analysis between variables and subgroups regarding the TTR (<65% or TTR ≥ 65%), and multivariate logistic regression analysis considering only variables that had achieved statistical significance in the univariate analysis. We estimated the odds ratio (OR) and 95% confidence intervals (95% CI). The results were considered statistically significant at a *p* -value < 0.05.

## Results

### Characteristics of the Patient Cohort

We identified adult patients taking VKA for VTE's long-term treatment, with a target INR of 2.0 to 3.0, from January 2010 to December 2013. In the acute phase, clinicians prescribed heparin in all patients, with 91.8% of patients receiving low-molecular-weight heparin (LMWH). A total of 171 patients (mean age 49.8 ± 16.6 years, 53% male) underwent a total of 804 INR measurements, with an average of 4.7 INR tests per patient. Half of the patients were over 50 years of age.

The main comorbidities were hypertension ( *n* = 60; 20.5%), a history of VTE ( *n* = 36; 21.1%), a history of cancer or on current treatment (25; 14.6%). VTE was unprovoked in 61 (35%) cases. Behçet's disease was present in 16 (9.4%) male patients.

### Time in Therapeutic Range and Factors Associated with Poor Quality of Anticoagulation

**Fig. 1:**
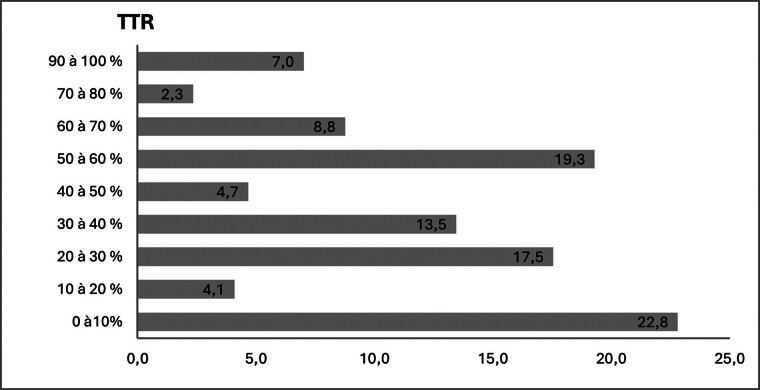
Distribution and frequency (%) of TTR values. TTR, time in therapeutic range.

The average TTR was calculated as 41.9 ± 32.9%. Of the sampled patients, 126 (74.3%) had a TTR below 65% ( Fig. 1 ).

We found no association between INR control and sex, primary diagnosis (PE or DVT), or comorbidities. The proportion of patients with low TTR was significantly higher in patients with a history of VTE [13.9 vs. 28.9%, *p* = 0.05], and the mean TTR was higher in unprovoked VTE than in provoked VTE [44.7 vs. 31.3%, *p* = 0.05], but the difference was no longer significant in multivariable analysis for those two variables ( Tables 1 – 2 ). In the multivariate logistic regression analysis, older age (≥65 years) and provoked VTE after surgery were found to be significantly associated with good anticoagulation control (TTR ≥ 65%) ( Table 2 ). Mean TTR determined by age was significantly higher in patients older than 65 years [39.2 ± 34.9 vs. 49.1 ± 34.9, *p* = 0.04], and the mean time spent below therapeutic INR range significantly higher (38.9 vs. 22.2%, *p* = 0.04) for those patients.

**Table 1:** Sociodemographic and clinical characteristics associated with poor TTR in patients with VTE on VKA

Independent variables	TTR < 65%	TTR ≥ 65%	Median TTR mean ± SD	*p*
**Age**				0.042
** <** **65 y**	105 (77.8)	30 (22.2)	39.2 ± 34.9	
** ≥65 y**	22 (61.1)	14 (38.9)	49.1 ± 34.9	
**History of VTE**				0.067
** Yes**	31 (86.1)	5 (13.9)	35.2 ± 30.7	
** No**	96 (71.1)	39 (28.9)	42.9 ± 33.4	
**Cancer history and treatment**				0.779
** Yes**	18 (72.0)	7 (28.0)	36.1 ± 33.5	
** No**	109 (74.7)	37 (25.3)	42.1 ± 32.9	
**Surgical history**				0.005
** Yes**	1 (20.0)	4 (80.0)	70.7 ± 41.3	
** No**	126 (75.9)	40 (24.1)	40.4 ± 32.4	

Abbreviations: SD, standard deviation; TTR, time in therapeutic range; VTE, venous thromboembolism.

**Table 2:** Results of univariate and multivariate analysis regression of predictor of good quality of anticoagulation

Variables	Univariate analysis			Multivariate analysis		
	OR	95% CI	*p*	OR	95% CI	*p*
Age ≥ 65 y	0.45	0.21–0.98	**0.045**	0.45	0.20–0.99	**0.048**
History of VTE	2.52	0.91–6.95	0.075	2.21	0.79–6.18	0.132
Surgical history	0.08	0.01–0.73	**0.025**	0.09	0.01–0.81	**0.032**

Abbreviations: CI, confidence interval; OR, odds ratio; VTE, venous thromboembolism.

We could not identify specific causes for low TTR (poor compliance, diet fluctuations, number of INR, co-medications).

We noted five cases of complications on treatment, including one case of clinically relevant hemorrhage, one case of PE, and three cases of recurrent DVTs. The TTR of these patients were, respectively, 0%, 59%, 35%, 35%, and 6%. The number of patients is too small to reach any statistical difference.

## Discussion

Literature lacks prior data on TTR values in Moroccan patients. In our sample of 171 patients with VTE, the mean TTR was 41.2%, with only 25.7% achieving optimal anticoagulation control (TTR > 65%). Notably, the history of surgery and age over 65 were the sole factors significantly associated with better control.

While the VTE burden in Africa remains undercharacterized, a review of 21 studies suggests a lower prevalence than in African-descendant populations abroad. 8 However, Maghreb populations share closer genetic and phenotypic profiles with Southern European cohorts. This is consistent with our previous audits in Moroccan hospitals, where the at-risk population mirrored European demographics. 9

While DOACs have transformed anticoagulation therapy, VKAs continue to be the most widely used agents in resource-constrained environments for VTE management and stroke prophylaxis in AF and valvular heart disease. Because of its narrow therapeutic window, multiple drug interactions, and individual variability in dose–response, the management of VKA therapy is a recognized challenge, which places patients at risk of bleeding if supratherapeutic and at risk of thromboembolic complications if subtherapeutic.

The most widely used method to assess the quality of anticoagulation is the TTR, as proposed by Rosendaal et al. 7 in 1993. TTR provides an estimation of the number of days the patient remained within the therapeutic interval (2.0–3.0) over time. In early 2008, Wan et al. performed a systematic review and linear regression analysis on the relationship between TTR and outcomes, which indicated a significant inverse correlation between TTR and complications (major bleeding and thromboembolism), even when adjusting for differences in patient cohort characteristics. 10 Recent data have confirmed this strong relationship between low TTR and the rate of bleeding or thromboembolic events; otherwise, a TTR above 65% has been reported to be associated with lower complication rates in AF. 3 4 11 12 13

For VTE, Mearns et al. suggest in a meta-analysis of 53 studies that for every 1% increase in TTR, recurrent VTE events are reduced by 0.46% per year (95% CI: 0.21–0.71%) and major bleeding events by 0.30% per year (95% CI: 0.04–0.55%). They also found that small improvements in INR control may have an even more significant effect on patients with a TTR < 65%. 3

Previous research has shown that TTR is slightly higher in patients with AF compared with patients with VTE. In a meta-analysis, the percentage of time in range (INR = 2–3) was 82.1% in AF patients versus 73.5% in VTE patients. 14 In a real-life setting, however, for VTE patients, the mean TTR is closer to 50%. 15 One explanation is that since patients with AF are usually on long-term VKA treatment, selection-to-continue bias will be more evident than in patients with VTE. Indeed, the quality of VKA treatment is highly dependent on the time interval since the start of treatment. A statistically significant lower TTR was seen in studies reporting a TTR that covers all INRs, including the first months, compared with studies reporting the TTR without the first month. 16 In the meta-analysis of INR control published by Erkens et al., patients with VTE had a TTR as low as 54%, with quality of control being highly dependent on the time interval since the start of treatment: 56% in studies including the first month in the calculation to 75% after the second month of treatment. 1 This difference is to be expected because of the difficulty reaching the therapeutic range in the initial treatment period and improvement in TTR during continued VKA treatment.

Individual factors have been recognized as being significantly related to poor anticoagulation control among VKA-treated patients; these include an increase in the number of medications administered by the patients, diabetes mellitus, hypertension, renal impairment, and neoplastic history. These conditions are independently associated with an increased risk of recurrent thrombotic events or major bleeding. 17 18 19 However, interpreting subanalyses from these reports remains challenging due to limited subgroup sizes and a lack of granular data.

Among these individual factors, advanced age plays a critical role. Aging involves pharmacokinetic and pharmacodynamic changes that often lead to a more unpredictable response to VKAs. 20 Consequently, numerous studies highlight the difficulty of achieving stable anticoagulation in elderly patients. 11 21 Contrary to these findings, yet consistent with other specific reports, 22 23 our study showed that younger patients (under 60 years) had poorer anticoagulation control than their older peers. Notably, in our cohort, patients over 65 did not receive more frequent monitoring (INR checks) compared with younger individuals. Possible explanations include poor adherence, social factors such as employment and alcohol use, poor and erratic dietary vitamin K intake, may justify this result. The younger age at VTE diagnosis observed in our study contrasts with typical reports. Although this may suggest underdiagnosis in older age groups, it does not truly reflect the current demographic trends in Morocco. Additionally, the high prevalence of Behçet's disease (9.4% in men)—a condition primarily affecting young adults—may partly explain the younger profile of our cohort. This likely reflects a context-specific VTE risk factor relevant to Morocco and other Maghreb countries. 24 Despite its prevalence, this variable did not emerge as a significant predictor of poor TTR in our multivariate analysis.

Cancer-associated thrombosis (CT) is also a therapeutic challenge. Our observed prevalence of 14.6% is consistent with the 11.6% rate reported across Africa, where disease patterns may differ from other global regions. 25 Patients with active cancer face a 2- to 3-fold higher risk of both recurrence and bleeding compared with those without malignancy. 26 27 Despite clinical guidelines clearly favoring LMWH or DOACs for CT, real-world data often reveal poor compliance, with VKAs still frequently prescribed. Hutten et al. previously demonstrated that cancer significantly decreases TTR in VTE patients treated with VKAs. 27 Even in the controlled environment of clinical trials, such as CLOT and CATCH, TTR values remained low (46 and 47%, respectively). 28 29 In our study, cancer patients exhibited a lower mean TTR compared with noncancer patients, although this difference did not reach statistical significance [42.1 ± 32.9 vs. 36.1 ± 33.5, *p* = NS].

Independent of a patient's clinical features, the regional location of medical care is a dominant determinant of TTR variation in global studies of VKA. In real-life patients, TTR is known to vary significantly across countries and health care settings. Connolly et al. assessed the median TTR in 526 centers and 15 countries and showed that there were wide variations across countries, from 46% in South Africa to 78% in Sweden. 2 Even after adjusting for all other significant features (but leaving out race because of co-linearity with region), geographic region's effect remained remarkable. 4 In VTE, a previous analysis suggested that patients being treated in Europe and the United Kingdom had a greater TTR compared with those in North America (+ 11%, *p* = 0.003). 3 Our findings complement other recent reports in lower-income countries like South Africa (TTR of 47%), 30 Turkey (mean TTR of 42.3 ± 18%), 31 or Tunisia (median TTR of 44.4%). 32 The same observation was made in Middle Eastern countries and East Asia (except Hong Kong and Singapore), in which the mean TTRs are around 45 to 50%, and with more than 70% of patients having poor anticoagulation. 33 34 The results are even worse in very low-income sub-Saharan countries; for example, in Namibia and Botswana or Uganda, the reported mean/median TTR lower than 40%. 30 35 36 Various factors may explain these differences. First, in high-income countries, validated dosing algorithms, 37 patient self-monitoring, 38 and anticoagulation clinics 39 40 frequently guide VKA dosing. Little work has been done on validated algorithms to guide dosing in low-income countries or the widespread use of other proven strategies to increase TTR. Second, there is a close relationship between lack of knowledge, low educational and socioeconomic levels, and poor anticoagulation control. Patients with inadequate health literacy were less likely to be aware of their anticoagulant treatment or their disorder, and limited health literacy was significantly associated with TTR < 50% (OR = 2.34, 95% CI [1.01, 5.46]), adjusting for sociodemographic data, health behaviors, and clinical characteristics. 41

In Western Europe, the UK, and North America, educational programs directed at patients are widely used and delivered by nurses or pharmacists. 42 43 Many findings support the efficacy of pharmacist intervention to improve TTR values in patients treated with VKAs. Frequent face-to-face appointments and intensive compliance support performed by trained pharmacists help to overcome social barriers. Recommendations such as dose adjustment and monitoring of drug–drug interactions, food interactions, and consumption of traditional medicine or supplements are even more easily assimilated. Clinical pharmacy intervention can also reduce work overload in physician-centered health care facilities. While the advantages of pharmacist-managed VKAs therapy in terms of anticoagulation control, safety, and mortality are still unclear, there is significantly better patient satisfaction. 43

Many authors have indicated that the used drug may determine the quality of treatment with VKA. Several studies have shown that long-acting VKAs (phenprocoumon and warfarin) provide more stable anticoagulation than those with a short half-life (acenocoumarol), 44 but results of other recent studies on the quality of treatment achieved with warfarin and acenocoumarol are conflicting, 45 46 and recently, Bryk et al. showed a similar quality of anticoagulation assessed by TTR in AF patients on acenocoumarol compared with warfarin in a Brazilian population. 47

Acenocoumarol is currently the only VKA available in Morocco. For patients with unstable anticoagulation, switching to warfarin or phenprocoumon could potentially optimize INR control. The dose–response variation of acenocoumarol is significantly influenced by genetic polymorphisms, particularly in CYP2C9 and VKORC1. These variants, along with CYP4F2, differ across ethnic groups and drive interindividual variability in dosing requirements of VKA. Indeed, a study of 217 Moroccan patients treated with acenocoumarol confirmed that VKORC1 and CYP2C9 polymorphisms are strongly associated with dose fluctuations. 48 While genotype-guided dosing algorithms have improved anticoagulation control and reduced overanticoagulation in high-income settings, 49 their implementation could be a key strategy for optimizing TTR in the Moroccan/African context.

Finally, the use of DOACs can overcome these difficulties encountered with VKAs. They have several advantages, including a more rapid onset than VKAs; they do not require heparin bridging, have fixed doses, are less susceptible to food and drug–drug interactions, and do not require monitoring. In seven randomized clinical trials for VTE, DOACs, and VKAs were comparable for recurrent VTE and fatal PE risk. Those trials demonstrated a 32 to 69% decreased risk of major bleeding than VKAs. 50 51 In resource-constrained environments, DOACs are often underutilized due to their price, yet they offer a critical solution for patients with labile INR despite good compliance. However, the economic burden of VKAs should not be assessed solely by acquisition cost; a broader perspective must include indirect expenses such as frequent INR monitoring, patient transportation and hemorrhagic complications. 52 Furthermore, the market entry of generic formulations may soon reduce barriers to access and improve affordability. As VKAs will likely remain widely used, optimizing anticoagulation control is a priority. Implementing “warfarin care bundles” tailored to the local environment represents a pragmatic strategy. By addressing management challenges in an integrated manner, such an approach is ideally suited to improve patient outcomes in the current Moroccan context.

We recognize some limitations in our study. First, the small sample size may have caused a lack of significance in some of the variables significantly associated with anticoagulation quality in previous studies. Also, we performed the study in a single center, and our findings need to be confirmed in other centers and another disorder (AF). Also, the inclusion of INR data from the first month of treatment may have reduced the percentage of TTR. Finally, as this study used a retrospective design, we cannot ensure that all data were correctly recorded in the past. Therefore, the results of the current study should be validated in a prospective cohort.

Despite these limitations, this is still the first study to evaluate the quality of anticoagulation in Morocco, and it provides good evidence of inadequate anticoagulation control in patients on VKA with VTE in clinical practice.

## Conclusion

Despite these limitations, this is still the first study to evaluate the quality of anticoagulation in Morocco, and it provides good evidence of inadequate anticoagulation control in patients with VTE in clinical practice on VKA. These data advocate for increased use of direct oral anticoagulants, notably anti-Xa agents, for better anticoagulation control.

## Notes

**What is Known about this Topic?**

- Few studies have primarily explored the quality of anticoagulation in patients with VTE. INR control is often poor in low-income countries. Variable altering the TTR in those patients have yet to be clearly determined.

**What Does This Paper Add?**

- This paper addresses a significant gap in literature by providing key data on VKA anticoagulation management for VT in an emerging country.
